# Effects of Supplementing Milk Replacer with Sodium Butyrate on Dairy Calves

**DOI:** 10.3390/ani14020277

**Published:** 2024-01-16

**Authors:** Anna Luiza Belli, Sandra G. Coelho, Joana P. Campolina, Luiz F. M. Neves, Hilton C. Diniz Neto, Camila S. Silva, Fernanda S. Machado, Luiz Gustavo R. Pereira, Thierry R. Tomich, Wanessa A. Carvalho, Suely de Fátima Costa, Mariana M. Campos

**Affiliations:** 1 Departamento de Zootecnia, Escola de Veterinária, Universidade Federal de Minas Gerais, Belo Horizonte 30161-970, MG, Brazil; annabelliac@gmail.com (A.L.B.); sandragesteiracoelho@gmail.com (S.G.C.); joana.campolina@yahoo.com.br (J.P.C.); luizneveszoo@yahoo.com.br (L.F.M.N.); hiltondinizneto@gmail.com (H.C.D.N.); 2 Empresa Brasileira de Pesquisa Agropecuária—Embrapa Gado de Leite, Juiz de Fora 36038-330, MG, Brazil; camilaszootecnia@gmail.com (C.S.S.); fernanda.machado@embrapa.br (F.S.M.); luiz.gustavo@embrapa.br (L.G.R.P.); thierry.tomich@embrapa.br (T.R.T.); wanessa.carvalho@embrapa.br (W.A.C.); 3 Departmento de Medicina Veterinária Preventiva, Universidade Federal de Lavras, Lavras 37203-202, MG, Brazil; sfcosta@ufla.br

**Keywords:** additive, butyrate supplementation, dairy calf, gastrointestinal tract development, growth promoter

## Abstract

**Simple Summary:**

Raising dairy calves poses a dual challenge for the dairy industry—economically and physiologically. The industry seeks innovative solutions that can not only reduce dependence on antibiotic treatments but also enhance the overall wellbeing, gastrointestinal tract development, and performance of young calves. Among the potential solutions, butyrate supplementation has emerged as a promising tool. To validate this hypothesis, the present study meticulously assesses the impact of butyrate supplementation on key parameters such as feed intake, gastrointestinal tract development, health, and performance of pre-weaned dairy calves. Furthermore, this investigation delves into the lasting effects of butyrate supplementation post weaning.

**Abstract:**

Diarrhea and respiratory diseases pose significant challenges in the rearing of pre-weaned calves, motivating the investigation of tools to improve gastrointestinal tract development, health, and overall performance in young calves. Consequently, the primary objective of this study was to assess the effectiveness of an additive incorporated into milk replacer to promote the development and health of the animals. Forty-six dairy calves were randomly assigned into two treatments: control (CON, *n* = 23; with 15 females and 8 males), and sodium butyrate (SB, *n* = 23; with 15 females and 8 males). The calves in the SB treatment group were supplemented with 4 g/d of unprotected sodium butyrate (Adimix, Adisseo, China), added to the milk replacer from 4 to 60 days of age. Water and starter were fed ad libitum. The study evaluated several parameters, including feed intake, nutrient digestibility, ruminal pH, ammonia and volatile fatty acids, blood metabolites (glucose, insulin-like growth factor type 1, urea, β–hydroxybutyrate), hemogram, health scores, performance, and feed efficiency. Bull calves were euthanized at 60 days of age for organ comparison, while heifer calves were assessed for carryover effects up to 90 days of age. Data were analyzed independently using linear mixed models using the nlme package in R, and the Artools package for non-parametric categorical outcomes. Although the feed intake and performance variables exhibited differences within weeks, no divergence was observed between treatment groups. Notably, a positive treatment-by-week interaction was identified for starter feed intake (*p* = 0.02) and total dry matter intake (*p* = 0.04) during pre-weaning for CON animals. Ruminal parameters, blood metabolites, and hemogram values such as glucose, urea, insulin-like growth factor type 1, mean corpuscular value, lymphocytes, and neutrophils displayed differences within weeks during the pre-weaning stage, but similar results within groups. No differences between supplemented and non-supplemented calves were found across nutrient digestibility, organ development, and histology. Regarding health scores, differences were noted within weeks for fecal and respiratory scores during the pre-weaning stage, and only the respiratory score during the post-weaning stage. Consequently, butyrate supplementation did not elicit improvements or negative effects in the body development or health status of dairy calves.

## 1. Introduction

Antimicrobial feed additives have been included in the diets of livestock since the 1950s, becoming a popular tool to improve performance, animal health, and farm profitability ever since. Nonetheless, the appearance of antimicrobial resistance over time became a dangerously common occurrence for both animals and humans [1,2], leading to the European Union’s ban on growth-promoting additives in 2006 [3]. As a result, researchers have attempted to find alternatives for enhancing animal performance while improving the herd’s health status and the food security of animal-derived products.

Diarrhea and respiratory diseases are the major causes of economic losses at the rearing stage on a dairy farm, sometimes accounting for more than 10% of the causes of mortality for young calves [4,5]. Short-chain fatty acids (SCFAs) naturally present in the mammalian gastrointestinal tract (GIT) have been pointed out as a promising option to reduce disease incidence in the early stages of a calf’s life [6]. Butyrate, one of the SCFAs found in milk and synthesized from the microbial fermentation of carbohydrates in the rumen and large intestines [7,8], plays a pivotal role in transepithelial fluid transport, the inhibition of mucosa inflammation, oxidative status, epithelial barrier defense, visceral sensibility, and intestinal motility [8,9] and can be used as an energetic source for rumen epithelial cells and colonocytes, which are vital for nutrient absorption [8,10].

Antibiotics administered during clinical disease episodes severely jeopardize the ability of intestinal cells to utilize butyrate because of disturbances to the intestinal microbiota [11]. This is especially critical for newborn calves, who have a high susceptibility to GIT disturbances and diseases and whose GIT is still underdeveloped. Recent research has indicated that this effect could be minimized by feeding exogenous sources of butyrate [12,13], which include sodium or calcium salts of butyric acid, usually encapsulated when added to solid feeds and non-encapsulated when added to a liquid diet [14]. These sources are designed to bypass the rumen to prevent butyrate’s degradation in the forestomach, thereby helping develop and mature the small and large intestines [15,16]. Several studies have demonstrated that the inclusion of sodium butyrate in a liquid diet improved the cell cycle of dairy calves, reducing apoptosis, stimulating cell proliferation and body antioxidant function, and strengthening the intestinal barrier and mucosal thickness [14,17,18].

Since encapsulated or non-encapsulated butyrate given to pre-weaned calves have improved GIT development and decreased the incidence of diarrhea [17,19,20,21], the objectives of this study were to evaluate the effects of supplementing pre-weaning calves with unprotected sodium butyrate through milk replacer (MR) on feed intake, ruminal pH, nitrogen ammonia (NH_3_-N) and volatile fatty acids (VFA) concentrations in rumen fluid, nutrient digestibility, blood concentrations of glucose, IGF-1, urea, and β–hydroxybutyrate (BHB), hemogram, health scores, performance, feed efficiency (FE), organ and GIT development during the pre-weaning phase, and the carryover effects during the post-weaning phase. The hypothesis was that supplementation with sodium butyrate in MR for dairy calves would improve their feed intake, nutrient digestibility, GIT development, health parameters, and performance.

## 2. Material and Methods

The experiment was conducted at the Embrapa Dairy Cattle, Minas Gerais, Brazil, from March to August 2018. All procedures were approved by the Ethics Committee of Embrapa Dairy Cattle, Brazil (protocol number 9078250118). Researchers were entrusted with the daily responsibilities of animal feeding, measurements, and health checks, ensuring the wellbeing and comfort of all calves throughout the study.

### 2.1. Animals, Housing, and Treatments

Thirty female and sixteen male, Holstein and Holstein × Gyr crossbred, dairy calves were enrolled in a randomized complete block design. After birth, calves were weighed and immediately moved to individual sand-bedded pens (1.25 × 1.75 m) tethered with 1.2 m chains, and allocated in an open-sided barn. All animals received 10% of their body weight in good-quality colostrum (Brix > 23%) and had their umbilical cord immersed in an iodine solution (10%) within two hours after birth. After the first colostrum feeding and until the third day of life, calves received 5 L/d of transition milk split into two meals (0800 and 1600 h), fed via a commercial milk feeder (Milkbar, Waipu, New Zealand). Blood was collected on the third day of life via jugular venipuncture into a tube without anticoagulant (Labor Import, Osasco, Brazil), and centrifuged at 3000× *g* for 10 min at room temperature (22–25 °C) to evaluate passive immune transfer using a Brix refractometer (Aichose refractometer, Xindacheng, Jiaozhou City, China). Only calves with a Brix value above 8.3% were enrolled in the study.

On the 4th of age, animals were blocked by sex and genetic composition and assigned to one of two treatments: control (CON, nothing added to the feed or to MR; *n* = 15 heifer and 8 bull calves); and unprotected sodium butyrate (SB; 4 g/d, Adimix, Adisseo, Nanjing, China) added to the MR; *n* = 15 heifer and 8 bull calves). The dose of butyrate was determined according to the levels of inclusion reported by other authors [10,19]. In every meal, the additive was diluted in the first 0.5 L of MR to ensure total ingestion of the product, resulting in supplementation of 2 g in the morning and 2 g in the afternoon.

All calves received 5 L per day of MR (Kalvolak, Nutrifeed, The Netherlands; Table 1) divided into two equal daily meals (0800 and 1600 h). The MR was mixed to provide 15% of total solids, providing 145.5 g of crude protein, and 105.8 g of fat, and offered to calves using a milk calf feeder bucket (Milkbar, New Zealand). Calf starter (Soymax Rumen pre-initial Flocculated, Total Alimentos, Três Corações, Brazil, Table 1) and water were individually offered ad libitum from the first day of life until weaning, at 60 days of age.

### 2.2. Feed Intake, Rumen Samples, Digestibility, Performance, and Nutritional Composition

Feed intake (MR, starter, water and, after weaning, corn silage) was measured daily using the difference between offers and refusals. Feed efficiency (FE) was calculated using the ratio between average daily gain (ADG) and dry matter intake (DMI) [22]. Samples of MR, starter, and corn silage (offered after weaning) were collected thrice a week and weekly pooled into individual subsamples and kept at −20 °C for further analysis.

Samples of ruminal fluid were collected on days 14, 28, 42, and 60 for all calves and on days 74 and 90 for heifers only, using an esophageal tube, four hours after the morning MR feeding. Ruminal fluid was strained through four layers of gauze to separate solid and liquid fractions. Rumen pH was immediately measured using a pH meter (Phmetro T-1000, Tekna, Araucária, Brazil), and two samples (10 mL) of ruminal fluid were obtained. One sample was acidified with 1 mL of 20% metaphosphoric acid, and the other with 2 mL of 50% sulfuric acid. The samples were stored at −20 °C for further VFA and NH_3_-N analyses. Samples used to test VFA concentrations were centrifuged at 1800× *g* for 10 min at room temperature (22–25 °C) and analyzed via high-performance liquid chromatography (Waters Alliance e2695 Chromatograph, Waters Technologies do Brazil LTDA, São Paulo, Brazil). Concentrations of NH_3_-N were quantified using a colorimetric distillation method [23]. Absorbance was measured at 630 nm (Thermo Fisher Scientific, Madison, WI, USA) after Kjeldahl distillation with magnesium oxide and calcium chloride according to Method 920.03 [24].

Digestibility was conducted only with the male calves. A rubber mat (WingFlex, Kraiburg TPE GmbH & Co., Ltd., Waldkraiburg, Germany) was placed atop the sand bed beneath each animal to facilitate the complete collection of feces over a period of five days. On the last day of the digestibility trial, animals were transferred to metabolic cages (1.50 m × 0.80 m; Intergado Ltd.a., Contagem, Brazil) for total urine collection and the last day of fecal collection. After the end of the collection (24 h), the urines’ total volume, weight, and density were measured. Two samples (50 mL) were collected from the total urine and kept at −20 °C for further analysis of urea. Samples of feces and the starter provided during the digestibility test were collected daily. After the end of the trial, a composite sample corresponding to the 5 days of digestibility was frozen at −20 °C. The apparent digestibility values of the nutrients (%) were determined using the amount consumed and the amount of each nutritional component recovered in the feces.

The pool of feed and fecal samples was ground through a 1 mm screen in a mill (model 3, Arthur H. Thomas Co., Philadelphia, PA, USA) before analysis. The dry matter content of the ground starter, corn silage, MR, and feces was oven-dried at 55 °C for 72 h (Method 934.01). Crude protein (CP) (Method 988.05), ether extract (EE) (Method 920.39), ash (Method 942.05), neutral detergent fiber (NDF), and acid detergent fiber (ADF) were determined using the methods [25].

For performance measurements, body weight (BW) was initially measured at birth and on day 3. After that, BW was measured once a week before the morning meal, using a portable scale (ICS 300, Coimma Comércio Indústria de Madeiras e Metalúrgica São Cristóvão Ltd.a., Dracena, Brazil). Wither height, hip height, hip width, and heart girth were measured at birth and every 7 d until the end of the experimental period. These measurements were taken on a flat surface using a portable hipometer (Walmur Instrumentos Veterinários Ltd.a., Porto Alegre, Brazil) and a metric tape.

### 2.3. Health Scores

Rectal temperature was evaluated daily at 0600h using a digital thermometer (Ombo Eletronics, iColor, modelo G-Tech, Shenzhen, China). Fecal [4] and respiratory scoring [26] was performed daily. A calf was considered to have diarrhea if the fecal score was 2 or 3. Diarrhea was considered severe when the fecal score was 3. Calves were treated with antibiotics only if the respiratory score was above 4 or if they had a fever for two consecutive days.

### 2.4. Blood Sampling and Weaning

Blood was drawn via jugular venipuncture using 10 mL tubes with anticoagulant and sodium fluoride at birth, before colostrum ingestion, and every 7 d thereafter, three hours after morning meals, for all animals and for the heifers until 90 d of age (Labor Import, Osasco, Brazil) for analysis of BHB, urea, and glucose concentrations. Also, every 14 days, 10 mL tubes containing heparin (Labor Import, Osasco, Brazil) were used to collect samples for IGF-1 concentrations. The samples were centrifuged at 3000× *g* for 10 min, at room temperature (22–25 °C), and two aliquots from each sample were individually stored frozen at −20 °C.

Serum concentrations of the BHB and urea were determined with an auto-analyzer (Cobas Mira Plus, Roche Diagnostic Systems, Risch-Rotkreuz, Switzerland) using commercial kits (Ranbut-D-3-Hidroxibutyrate, Randox Laboratories Ltd., Antrim, UK; Urea UV, Kovalent do Brazil Ltd.a., Bom Retiro São Gonçalo, Brazil). The concentration of plasma glucose was measured via a Spectrophotometer EON microplate (Biotek Instruments Inc., Winooski, VT, USA) using an enzymatic colorimetric method (Kovalent do Brazil Ltd.a., Rio de Janeiro, Brazil). Plasma concentrations of IGF-1 were determined using a chemiluminescence assay (Immulite 2000 Systems 1038144, IGF-1 200, Siemens Healthcare Diagnostics Products Ltd., Llanberis, Gwynedd, UK).

At days 0, 30, and 60, blood from all animals was sampled in 2 mL tubes containing EDTA (Labor Import, Osasco, Brazil), stored at 4 °C, and immediately sent to the laboratory for hemogram analysis.

At 60 d of age, heifer calves were weaned, kept in the experiment and, after weaning, at d 61, started receiving corn silage as part of the diet (Table 1). The amount of corn silage provided was enough to assure at least 10% of orts, and the starter intake was limited to 3.0 kg heifer/d, fed divided into two meals. Water continued to be offered ad libitum.

### 2.5. Internal Organ Weight, Gastrointestinal Tract Development, and Histology

At day 60 of age, male calves were euthanized to evaluate the weight of organs and tissue sampling, using the procedures recommended by the Brazilian Federal Veterinary Medicine Council [27] Briefly, the chose method consisted of the administration of sedative (0.05 mg/kg BW of xylazine intramuscularly) followed by the administration of intravenous barbituric acid (20 mL of pentobarbital).

The tongue, lungs with trachea, heart, liver, spleen, kidneys, mesentery, omental and peri-kidney fat were weighed and discarded. The rumen–reticulum, omasum, abomasum, small and large intestines, and bladder were weighted, full of biological content, and emptied. All variables were evaluated in proportion to the weight of the empty body. The length of the small and large intestines was measured using a metric tape.

Samples of 9 cm^2^ (3 × 3 cm) were obtained from the rumen (dorsal sac, ventral sac), omasum, abomasum, and portions of the small and large intestines (duodenum, ileum, and colon). The samples were fixed in formalin, and after fixation, they were kept in an ethanol solution (alcohol 70%) until the analysis was performed. The samples were incorporated into paraffin blocks, being sectioned using a manual microtome (Olympus, Tokyo, Japan) in sections 5 μm thick. For morphometric analysis, the slides were stained with hematoxylin–eosin. The images were captured with the aid of a microscope (Olympus CX31, Tokyo, Japan), coupled with a camera (Olympus OSIS SC30, Tokyo, Japan), using the Cell-B software version 3.4 (Olympus, Tokyo, Japan). Papillary height (μm) and papillary area (μm^2^) were measured in regions of the rumen’s ventral and dorsal sac, and the omasum. Villus height (μm) and villus area (μm^2^) were measured in regions of the duodenum and ileum. Fossette depth (μm) and gastric gland depth (μm) were measured in the abomasum. The crypt depth (μm) was also measured in regions of the duodenum, ileum, and colon.

Mitotic index (MI) was determined in the epithelium of the rumen (ventral and dorsal sac) and omasum, using an optical microscope (Olympus OSIS SC30, Tokyo, Japan) with 400× enlargement. Approximately 2000 cells from the epithelium basal layer were counted, including those presenting mitotic figures in the nucleus. The index was calculated using the quotient between the number of nuclei in division and the total of nuclei counted [28]. The cell proliferation in regions of the abomasum, duodenum, ileum, and colon was determined by counting the number of mitotic figures in the epithelium of gastric and intestinal glands, in 10 fields, using a 400× enlargement.

### 2.6. Statistical Analysis

The data were analyzed using the software R version 4.3.1 [29]. The pre-weaning (1–60 d) and post-weaning (61–90 d) data were analyzed separately. A randomized complete block design with repeated measures was used to test the effect of sodium butyrate on each outcome measured. Treatment, week, and the interaction of treatment and week were used as fixed effects. The effect of calf within treatment was included in the models to account for individual variability.

Feed intake, ruminal parameters, digestibility, rectal temperature, blood parameters, body measurements, performance, and feed efficiency, were analyzed using the linear mixed model (package: nlme) [30], including calf as the random term, treatment, week, and their interaction as fixed variables.

Respiratory and fecal score were analyzed using a non-parametric aligned rank transformation methodology implemented via the software package ARTool version 0.11.1 [31]. Differences between averages of treatments were evaluated using the Fisher test, and weekly and interactions weekly × treatment were evaluated through the Tukey test.

Variables with a single measurement during the study, such as final and initial BW, organ/viscera weight and GIT development were analyzed using the linear mixed model (package: nlme) [30], including calf as the random term and treatment as a fixed variable. All outcomes underwent testing for homoscedasticity and normality using Bartlett and Shapiro tests to satisfy the necessary assumptions of this model. Significance was asserted at *p* ≤ 0.05.

## 3. Results

### 3.1. Feed Intake, Rumen Parameters, Performance, and Feed Efficiency

In the current study, milk replacer intake, water intake, body measurements, feed efficiency, and average daily gain were not different (*p* > 0.05) among treatments during all the evaluation periods (Table 2 and Table 3). Water intake was higher (*p* = 0.01) for CON animals compared to SB animals in the post-weaning period (Table 2). There was a significant effect of week (*p* < 0.05) on feed intake and performance variables. The milk replacer intake was smaller in weeks 2 and 3, while the average dairy gain, starter, and water intakes, as well as the total DMI showed significant increases in the subsequent weeks both during pre- and post-weaning *(p* < 0.001, Table 2). There was a positive treatment-by-week interaction for starter feed intake and total DMI during pre-weaning (*p* < 0.05, Table 2), whereas in weeks 3 to 5 (*p* < 0.05, Table 2), CON animals ate, on average, 44 g more starter than SB animals. As for the total DMI, the CON animals presented higher intake values compared to SB between weeks 4 and 7, eating, on average 5.7% more than SB calves.

For ruminal parameters, there were no differences among treatments during the pre- and post-weaning periods (*p* > 0.05, Table 4). The ruminal NH_3_-N and VFA were influenced by the week during the pre-weaning period, mirroring the observed pattern in intake (*p* < 0.001, Table 4). The cecum concentrations of VFA, evaluated only for the bull calves at 60 days of age, did not show any differences among treatments (*p* > 0.05, Table 4).

### 3.2. Digestibility, Comparative Slaughter, and Histology

The digestibility and nitrogen balance performed with the bull calves at the end of the pre-weaning period were not different (*p* > 0.05, Table 5) among treatments. As for comparative slaughter, there were no differences regarding organ weight, intestine length, or histological parameters among the CON and SB calves (*p* > 0.05, Table 6 and Table 7).

### 3.3. Blood Parameters and Health Scores

No differences were found regarding the evaluated blood parameters (*p* > 0.05, Table 8 and Table 9). A week effect was observed on urea, glucose, and IGF-1 concentration, and mean corpuscular volume, absolute lymphocytes, and segmented neutrophils during the pre-weaning phase (*p* < 0.05, Table 8 and Table 9), and on glucose in the post-weaning period (*p* < 0.05, Table 8). The IGF-1 values increased over time. In contrast, glucose levels followed a divergent pattern, decreasing, and this trend persisted post weaning. Urea exhibited higher values in the third and fourth weeks but decreased thereafter. The mean corpuscular volume and neutrophils exhibited a decreasing trend over time. In contrast, lymphocyte levels showed an increase throughout the weeks.

While assessing the impact of sodium butyrate on clinical responses, no treatment effect was observed on the health scores of calves. However, a significant week effect was identified for fecal scores (*p* < 0.001) during the pre-weaning period, particularly in weeks 2 and 3. Additionally, respiratory scores showed significant variations during both the pre- and post-weaning periods, specifically in weeks 3, 4, and the 9th week (one week after weaning) (*p* < 0.001, Table 10). No diarrhea treatments were administered for SB animals, while for the CON group, two animals had to be treated with antibiotics, non-steroidal anti-inflammatory, and oral hydration fluids, with an average of 2.0 ± 1.4 days in treatment.

## 4. Discussion

The use of additives in dairy management has increased widely, especially of products that could be a potential alternative to antimicrobial growth promoters and that can assist animal development [2]. Previous work suggested that the inclusion of exogenous butyrate could be an alternative to help GIT development in calves [6,12] and improve animals’ health [13]. However, the type of exogenous butyrate, such as protected sources, could preserve it from ruminal microbial action. However, even when feeding unprotected sources of butyrate in the MR, feeding it with a milk calf feeder bucket facilitates ruminal bypass and increases butyrate activity in the GIT [14]. Additionally, besides the dose and the source type of butyrate used, the duration of administration can exert a major impact on the practical results of butyrate supplementation [9,32]. Even so, the route, dosage, and source of butyrate still vary a lot among researchers. In the present study, an unprotected source of butyrate was chosen to be fed in the liquid diet. The chosen dosage was like the average reported by other authors [10,17,32,33,34,35]. However, the period of its supplementation through MR was longer than most of what has been published so far, with additional evaluation of outcomes and carryover effects. Thus, this study aimed to evaluate the supplementation in MR of unprotected sodium butyrate for pre-weaned dairy calves and its impact on performance, gut development, and health.

### 4.1. Feed Intake, Rumen Parameters, Performance, and Feed Efficiency

Contrary to what was expected, sodium butyrate supplementation did not exert a positive effect on the feed intake, weight gain, performance, and digestibility of the supplemented calves. These findings corroborate others that did not demonstrate increases in performance [36,37], DMI [19,37,38], or digestibility [34,36,39] through butyrate supplementation. Reinforcing our findings, previous works found no differences in ADG and biometrical evaluations such as WH, HG, and RH between supplemented and control animals when evaluating the same product and dosage used in this present work [35]. As for nutrient digestibility, it is also important to mention that previous findings showed an increase in the digestibility of some components like fat, ash, and calcium for calves fed with a soy-based MR [40]. The MR used in the present trial had a high percentage of protein derived from milk and wheat protein. Therefore, the diet protein source could also have impacted the results. Thus, not all supplementation conditions seem to favor the use of butyrate as an additive for calves, and other experiments using it as a growth promoter supplement for this animal category are not consistent with body weight gain and performance results [16,41,42]. Thus, although the digestibility was similar between treatment groups, the positive treatment-by-week interaction for starter intake, and consequently the total DMI for the CON group, suggest a possible detrimental effect of butyrate supplementation. Therefore, given the different routes of supplementation, different doses, and differences in additive protection or not, it is still difficult to affirm the possibility of using sodium butyrate as an additive to increase performance, intake, and digestibility in dairy calves.

Most of the butyric acid produced in the rumen as a result of the feed fermentation by microorganisms, especially fibrous carbohydrates, is metabolized in the rumen epithelium cells and transformed into BHB [43]. This metabolization provides energy for these cells in acetyl-CoA and decreases the load of butyric acid to the liver and peripheral tissues [44]. The effects of time on the concentrations of urea, butyric acid, and C2:C3 ratio in rumen content can be directly explained by the increase in DMI as calves grow, increasing the availability of substrates for ruminal fermentation. Since no differences were found for ruminal parameters and development, no differences in blood parameters were expected.

### 4.2. Digestibility, Comparative Slaughter, and Histology

Dietary butyrate is mostly metabolized by the ruminal and gut epithelium, providing energy to the local cells and increasing mitotic indices and cellular growth [43]. Since, in the present experiment, butyrate was administered through the liquid diet, therefore bypassing the rumen, changes in GIT development were expected. However, the histological results found herein indicate that the supplementation strategy used was ineffective at promoting the beneficial effects of butyrate on the cellular cycle at the intestinal level of the calves. Previous work reported that calves fed rumen-protected butyrate in MR before weaning had increased ruminal papilla length and width [19]. Other research reported that the supplementation of sodium butyrate, even produced through the MR, may increase the concentration of butyrate in the rumen [45], probably due to an impaired closure of the esophageal leak [46,47]. This effect on the ruminal papillae was not observed in our study using unprotected sodium butyrate. In the present study, nipple buckets were used to feed MR to the calves, which may have resulted in less milk being directed to the rumen. Previous studies comparing protected and unprotected sodium butyrate in the starter found that the unprotected source increased the papilla surface in the rumen and the protected source increased mucosa thickness in the abomasum and tended to increase the whole organ weight [13]. A similar study, using the same commercial sodium butyrate and the same dose as in the present study, found that animals that were euthanized earlier, at 30 days of age, had higher ruminal and duodenum development [35]. Thus, the addition of supplements could potentially enhance health during the initial weeks of life, particularly in light of the occurrence of neonatal diseases.

### 4.3. Health Scores

Lastly, since one of the hypotheses of the present work was that sodium butyrate would influence health parameters, health scores were evaluated daily to check if the additive would have a positive impact on these outcomes. Nevertheless, no difference was found for the health results, contradicting the hypothesis that the sodium butyrate could help overcome neonatal disease challenges, as well as helping to regulate the inflammation process and status. Similar to the present study, earlier studies did not find consistent effects from supplementing butyrate in the MR on the health status of evaluated calves [41]. Using the same commercial additive as the current research, earlier findings reported lower diarrhea morbidity and recurrence; however, there is no information on the type of milk given in the liquid diet, diet management, facilities, and time of the year the trial was conducted [35]. Thus, these differences in the health status of supplemented animals could be due to management challenges. Since diarrhea is a clinical sign of several diseases frequently caused by bacterial, protozoans, and viral pathogens, we can expect to find controversial results in the literature. Unfortunately, little is known about sodium butyrate’s effects on the ruminant immune response. New experiments should be performed, especially focusing on the first 30 days of life, to verify the supplementation effects on the immunomodulation, performances, and physiology of calves to overcome neonatal disease.

## 5. Conclusions

The absence of significant changes in response to the 4g dose of sodium butyrate (0.5% of MR DM) in this study highlights the nuanced nature of animal physiology. This may suggest that the intervention did not elicit the anticipated physiological responses or that the physiological systems demonstrated stability and adaptability. The present findings contribute to a comprehensive understanding, guiding future research, and acknowledging the complexity of biological systems. These insights underscore the need for refined approaches, optimal dosages, and a holistic interpretation of experimental outcomes in the study of animal physiology.

## Figures and Tables

**Table 1:** Nutrient composition (% DM ± SD) of milk replacer (MR), starter, and corn silage.

Item	MR ^1^	Starter ^2^	Corn Silage
DM (%)	96.0 ± 0.4	86.7 ± 0.7	36.1 ± 3.1
CP (% of DM)	19.4 ± 0.5	17.1 ± 0.5	7.9 ± 0.7
Ether extract (% of DM)	14.1 ± 0.6	3.9 ± 1.2	4.3 ± 0.5
Organic matter (% of DM)	9.7 ± 0.2	7.2 ± 1.5	6.0 ± 1.1
NDF (% of DM)	-	22.1 ± 2.9	46.1 ± 4.1
ADF (% of DM)	-	10.6 ± 0.9	28.9 ± 3.5
Metabolizable energy (Mcal/kg DM) ^3^	3.6 ± 0.1	2.8 ± 0.0	2.4 ± 0.1

^1^ Powder integral milk, wheat isolated protein, acidifying additive, whey, coconut oil, palm oil, vitamin A, Vitamin D3, Vitamin E, Vitamin C. ^2^ Basic composition: oats (rolled grains), calcitic limestone, sodium chloride, corn gluten meal, defatted corn germ, wheat bran, soybean meal, rice hulls, kaolin, molasses, flocculated corn, ground corn, corn grain, alfalfa hay, monensin, citrus pulp, dried sugarcane yeast, whole toasted soybean, sodium selenite, copper sulfate, manganese sulfate, cobalt sulfate, iron sulfate, zinc sulfate, calcium iodate, vitamin A, vitamin B1, vitamin B12, vitamin B2, vitamin B6, vitamin C, vitamin D3, vitamin E, vitamin K, niacin, pantolenic acid, folic acid, biotin, propionic acid, caramel aroma, milk aroma, and probiotic additive. ^3^ Metabolizable energy was calculated from NRC (2001).

**Table 2:** Feed intake, feed efficiency, and performance of dairy calves in the control group (CON, n = 23) and supplemented group (SB, *n* = 23) in the pre-weaning period, and the control (CON, *n* = 15) and supplemented (SB, *n* = 15) groups in the post-weaning period.

Item	Treatment ^1^		SEM	*p*-Value ^2^		
	CON	SB		T	W	T × W
Feed intake—pre-weaning						
Starter (g of DM/day)	259	223	171	0.88	<0.001	0.02
Milk replacer (g of DM/day)	729	726	76	0.67	<0.001	0.85
Water (mL)	1176	1271	667	0.48	<0.001	0.40
Total DMI (g/day)	987	945	186	0.88	<0.001	0.04
Performance—pre-weaning						
ADG (g/day)	527	509	15	0.62	<0.001	0.69
Feed efficiency	0.5	0.5	0.02	0.45	<0.001	0.85
Feed intake—post-weaning						
Starter (g of DM/day)	1843	1468	342	0.09	<0.001	0.59
Corn silage (g of DM/day)	145	128	66	0.27	<0.001	0.86
Water (mL/day)	5695	4848	1310	0.01	<0.001	0.85
Total DMI (g/day)	1957	1312	345	0.15	<0.001	0.35
Performance—post-weaning						
ADG (g/day)	880	796	0	0.58	<0.001	0.31
Feed efficiency	0.4	0.4	0.03	0.69	0.55	0.37

^1^ CON = control; SB = 4 g of sodium butyrate (Adimix, Adisseo, China) supplemented from 4 to 60 d of age; ^2^ T = treatment effect; W= week effect, T × W = treatment by week interactions.

**Table 3:** Body measurements of control (CON, *n* = 23) and supplemented (SB, *n* = 23) groups in the pre-weaning period and the control (CON, *n* = 15) and supplemented (SB, *n* = 15) groups in the post-weaning period.

Item		Treatment ^1^		SEM	*p*-Value ^2^
		CON	SB		T
At Birth	Weight (kg)	33.1	32.2	0.61	0.57
	Wither height (cm)	68.3	67.7	0.38	0.56
	Rump height (cm)	71.3	70.8	0.39	0.67
	Rump width (cm)	17.0	16.9	0.16	0.72
	Heart girth (cm)	74.6	72.9	0.46	0.16
Weaning	Weight (kg)	64.8	62.7	1.07	0.47
	Wither height (cm)	80.6	80.2	0.41	0.71
	Rump height (cm)	83.7	83.9	0.40	0.89
	Rump width (cm)	21.6	21.5	0.13	0.77
	Heart girth (cm)	91.6	91.5	0.45	0.99
Final	Weight (kg)	82.7	82.9	2.32	0.33
	Wither height (cm)	86.0	85.5	0.98	0.16
	Rump height (cm)	89.0	87.9	0.98	0.89
	Rump width (cm)	23.8	23.1	0.45	0.17
	Heart girth (cm)	102.0	98.0	1.55	0.14

^1^ CON = control; SB = 4 g of sodium butyrate (Adimix, Adisseo, China) supplemented from 4 to 60 d of age; ^2^ T = treatment effect.

**Table 4:** Rumen parameters of the control (CON, *n* = 23) and supplemented (SB, *n* = 23) groups in the pre-weaning period, and cecum parameters of 60-day bull calves in the control (CON, *n* = 8) and supplemented (SB, *n* = 8) groups.

Item	Treatment ^1^		SEM	*p*-Value ^2^		
	CON	SB		T	W	T × W
Pre-weaning						
pH	6.1	6.2	0.47	0.53	0.16	0.72
Ammonia-N (mg/dL)	11.4	12.8	0.07	0.31	<0.001	0.35
Volatile fatty acids (mmol/L)						
Acetic (C2)	27.8	25.5	7.78	1.0	<0.001	0.57
Propionic (C3)	15.0	16.4	7.18	0.72	<0.001	0.90
Butyric (C4)	3.8	3.6	1.55	0.52	<0.001	0.35
C2:C3	1.9	1.9	0.54	0.74	<0.001	0.65
Post-weaning						
pH	6.2	6.1	0.53	0.44	0.09	0.82
Ammonia-N (mg/dL)	11.0	9.0	9.17	0.20	0.75	0.56
Volatile fatty acids (mmol/L)						
Acetic (C2)	37.7	38.6	9.50	0.33	0.64	0.28
Propionic (C3)	27.7	27.9	7.04	0.52	0.39	0.48
Butyric (C4)	5.6	6.3	1.61	0.91	0.39	0.76
C2:C3	1.4	1.4	0.20	0.60	0.32	0.58
Cecum						
Acetic (C2)	21.3	20.5	0.33	0.90	-	-
Propionic (C3)	13.1	12.3	0.41	0.84	-	-
Butyric (C4)	2.2	3.0	0.07	0.14	-	-
C2:C3	1.7	1.7	0.07	0.63	-	-

^1^ CON = control; SB = 4 g of sodium butyrate (Adimix, Adisseo, China) supplemented from 4 to 60 d of age; ^2^ T = treatment effect; W= week effect, T × W = treatment by week interactions. The “-” means that this outcome was not tested W or T × W.

**Table 5:** Nutrient apparent digestibility, from 54 to 59 days of age, of bull dairy calves in the control (CON, *n* = 8) and supplemented (SB, *n* = 8) groups.

Item	Treatment ^1^		SEM	*p*-Value ^2^
	CON	SB		T
Dry matter (g/day)	877	889	4.02	0.45
Organic matter (g/day)	919	911	3.01	0.57
Crude protein (mg/day)	921	932	1.98	0.60
Ether extract (mg/day)	957	965	1.58	0.53
Ingested nitrogen (g/kg of MW ^3^/day)	2.1	2.0	0.03	0.79
Fecal nitrogen/(g/kg of MW ^3^/day)	0.2	0.2	0.01	0.66
Urine nitrogen/(g/kg of MW ^3^/day)	0.4	0.4	0.02	0.87
Retained nitrogen (g/kg of MW ^3^/day)	1.6	1.5	0.01	0.79

^1^ CON = control; SB = 4 g of sodium butyrate (Adimix, Adisseo, China) supplemented from 4 to 60 d of age; ^2^ T = treatment effect. ^3^ MW = metabolic weight.

**Table 6:** Results of comparative euthanasia and measurement of organs of bull dairy calves in the control (CON, *n* = 8) and supplemented (SB, *n* = 8) groups.

Item	Treatment ^1^		SEM	*p*-Value ^2^
	CON	SB		T
Live body weight (kg)	65.4	67.7	0.77	0.52
Empty carcass (kg)	53.3	53.8	0.57	0.84
Tong (kg)	0.47	0.55	0.002	0.77
Lungs and trachea (kg)	2.49	2.28	0.08	0.51
Heart (kg)	0.77	0.77	0.003	0.97
Spleen (kg)	0.72	0.74	0.02	0.86
Liver (kg)	2.57	2.6	0.09	0.87
Pancreas (kg)	0.05	0.06	0.003	0.57
Kidneys (kg)	0.55	0.65	0.029	0.31
Bladder (kg)	0.06	0.05	0.002	0.54
Reticulum–rumen (kg)	1.08	1.21	0.35	0.39
Omasum (kg)	0.22	0.20	0.06	0.68
Abomasum (kg)	0.35	0.35	0.09	0.90
Small intestine (kg)	1.84	1.73	0.13	0.31
Small intestine length (m)	21.97	22.58	1.34	0.66
Large intestine (kg)	0.63	0.55	0.08	0.17
Large intestine length (m)	3.48	3.23	0.47	0.22
Omental fat (kg)	0.38	0.36	0.003	0.12
Peri-kidney fat (kg)	0.42	0.55	0.009	0.11
Mesentery + omental fat (kg)	0.46	0.48	0.007	0.82

^1^ CON = control; SB = 4 g of sodium butyrate (Adimix, Adisseo, China) supplemented from 4 to 60 d of age. ^2^ T = treatment effect.

**Table 7:** Results of the histology analysis of the gastrointestinal organs of bull calves in the control (CON, *n* = 8) and supplemented (SB, *n* = 8) groups.

Item		Treatment ^1^		SEM	*p*-Value ^2^
		CON	SB		T
Rumen—ventral sac	Cell proliferation	11.21	9.75	0.67	0.63
	Total cells	2011.12	2010.75	0.66	0.63
	Mitotic index	0.005	0.005	0.0003	0.67
	Papillary height (mm)	1.24	1.49	0.039	0.18
	Papillary area (mm)	9.30	6.50	0.83	0.45
Rumen—dorsal sac	Papillary height (mm)	2.68	2.35	0.006	0.27
	Papillary area (mm)	4.60	3.70	0.01	0.15
Omasum	Cell proliferation	18.50	14.50	0.87	0.33
	Total cells	2019.0	2014.5	0.87	0.32
	Mitotic index	0.007	0.009	0.0004	0.35
	Papillary area (mm)	0.13	0.09	0.01	0.25
	Papillary height (mm)	0.13	0.22	0.002	0.34
Abomasum	Cell proliferation	8.56	10.12	0.36	0.35
	Fossette depth (mm)	0.27	0.22	0.006	0.08
	Glandular depth (mm)	0.15	0.12	0.003	0.20
Duodenum	Cell proliferation	23.3	22.8	0.77	0.87
	Villus height (mm)	0.37	0.41	0.01	0.43
	Villus area (mm)	0.51	0.65	0.012	0.51
	Crypt depth (mm)	0.31	0.28	0.05	0.27
Ileum	Cell proliferation	40.5	40.4	2.54	0.99
	Villus height (mm)	0.28	0.28	0.007	0.82
	Villus area (mm)	0.32	0.35	0.001	0.65
	Crypt depth (mm)	0.28	0.27	0.005	0.64
Colon	Cell proliferation	13.0	10.10	0.71	0.39
	Crypt depth (mm)	0.37	0.35	0.008	0.48

^1^ CON = control; SB = 4 g of sodium butyrate (Adimix, Adisseo, China) supplemented from 4 to 60 d of age; ^2^ T = treatment effect.

**Table 8:** Pre- and post-weaning blood concentrations of β–hydroxybutyrate (BHB), urea, glucose, and insulin-like growth factor type I (IGF-1) for the control (CON, *n* = 23) and supplemented (SB, *n* = 23) groups in the pre-weaning period and the control (CON, *n* = 15) and supplemented (SB, *n* = 15) groups in the post-weaning period.

Item	Treatment ^1^		SEM	*p*-Value ^2^		
	CON	SB		T	W	T × W
Pre-weaning						
BHB (mmol/L)	0.2	0.1	0.03	0.65	0.17	0.92
Urea (mg/dL)	11.4	12.2	4.0	0.13	<0.001	0.37
Glucose (mg/dL)	100.4	104.9	12.4	0.21	<0.001	0.27
IGF-1 (ng/mL)	94	85.4	34.6	0.89	<0.001	0.36
Post-weaning						
BHB (mmol/L)	0.3	0.3	0.02	0.68	0.13	0.59
Urea (mg/dL)	22.5	32.5	5.6	0.23	0.29	0.46
Glucose (mg/dL)	88.5	90.7	7.5	0.21	<0.001	0.19
IGF-1 (ng/mL)	160.7	179.7	21.6	0.16	0.18	0.12

^1^ CON = control; SB = 4 g of sodium butyrate (Adimix, Adisseo, China) supplemented from 4 to 60 d of age; ^2^ T = treatment effect; W= week effect, T × W = treatment by week interactions.

**Table 9:** Hemogram of samples collected on day 0, 30, and 60, of dairy calves in the control (CON, *n* = 23) and supplemented (SB, *n* = 23) groups in the pre-weaning period.

Item	Treatment ^1^		SEM	*p*-Value ^2^		
	CON	SB		T	W	T × W
Erythrocytes (×10^6^/µL)	7.8	7.8	1.1	0.93	0.30	0.81
Hemoglobin (q/dL)	10.8	10.9	1.5	0.93	0.69	0.69
Mean corpuscular volume (fL)	44.6	45	4.1	0.61	0.002	0.62
Packet cell volume (%)	34.7	35	4.8	0.83	0.75	0.73
Mean corpuscular hemoglobin concentration (%)	31.3	31.2	1.1	0.55	0.96	0.57
Platelets (×10^3^/µL)	389	376.1	107.4	0.32	0.24	0.12
Total leucocytes	10,357.2	10,441.8	2330.0	0.97	0.26	0.65
Absolute monocytes (/µL)	438.7	519.4	376.0	0.67	0.19	0.05
Absolute lymphocytes (/µL)	4872.6	5262.3	1450.0	0.23	<0.001	0.62
Segmented neutrophils (/µL)	4920.8	4534.1	1920.0	0.19	<0.001	0.48
Band neutrophils (/µL)	29.2	42.4	5.9	0.17	0.22	0.54
Eosinophils (/µL)	86.3	72.4	25.7	0.64	0.75	0.03
Plasmatic protein (g/dL)	6.0	6.0	0.7	0.36	0.19	0.30

^1^ CON = control; SB = 4 g of sodium butyrate (Adimix, Adisseo, China) supplemented from 4 to 60 d of age. ^2^ T = treatment effect; W = week effect, T × W = treatment by week interactions.

**Table 10:** Health scores during the pre- and post-weaning period of dairy calves in the control (CON, *n* = 23) and supplemented (SB, *n* = 23) groups in the pre-weaning period and the control (CON, *n* = 15) and supplemented (SB, *n* = 15) groups in the post-weaning period.

Item	Treatment ^1^		SEM	*p*-Value ^2^		
	CON	SB		T	W	T × W
Pre-weaning						
Fecal score	0.49	0.52	0.02	0.85	<0.001	0.96
Respiratory score	0.68	0.66	0.04	0.64	<0.001	0.67
Days with respiratory score > 4	1.65	1.26	0.18	0.36	-	-
Days with fever	1.39	1.39	0.2	0.81	-	-
Days with diarrhea	8.78	8.96	0.75	0.99	-	-
Days with severe diarrhea	3.22	2.61	0.27	0.35	-	-
Post-weaning						
Fecal score	0.04	0.05	0.007	0.36	0.61	0.89
Respiratory score	1.10	1.01	0.04	0.47	<0.001	0.69
Days with respiratory score > 4	0	0	-	-	-	-
Days with fever	0.52	0.49	0.13	0.18	-	-

^1^ CON = control; SB = 4 g of sodium butyrate (Adimix, Adisseo, China) supplemented from 4 to 60 d of age. ^2^ T = treatment effect; W = week effect, T × W = treatment by week interactions. The “-” means that this outcome was not tested W or T × W.

## Data Availability

Data are contained within the article.
